# Preserved Ratio Impaired Spirometry in Low- and Middle-Income Countries: An Emerging Cardiopulmonary Phenotype and Cardiovascular Risk—A Narrative Review

**DOI:** 10.3390/life16050735

**Published:** 2026-04-28

**Authors:** Ramona Cioboata, Silviu Gabriel Vlasceanu, Maria-Loredana Tieranu, Denisa Maria Mitroi, Eugen Nicolae Tieranu, Gabriela Marina Andrei, Mara Amalia Balteanu, Anca Lelia Riza, Mihai Olteanu

**Affiliations:** 1 Department of Pneumology, University of Medicine and Pharmacy, 200349 Craiova, Romania; ramona_cioboata@yahoo.com (R.C.); gabriela.andrei@umfcv.ro (G.M.A.); mihai.olteanu@umfcv.ro (M.O.); 2 Department of Pneumology, Victor Babes University Hospital, 200349 Craiova, Romania; 3 Department of Microbiology, “Carol Davila” University of Medicine and Pharmacy, 050474 Bucharest, Romania; silviu.vlasceanu@drd.umfcd.ro; 4 Department of Thoracic Surgery, Marius Nasta Pneumology Institute, 050159 Bucharest, Romania; 5 Department of Obstetrics and Gynecology, Emergency County Hospital Craiova, 200642 Craiova, Romania; 6 Doctoral School, University of Medicine and Pharmacy, 200349 Craiova, Romania; 7 Department of Internal Medicine-Cardiology, University of Medicine and Pharmacy Craiova, 200349 Craiova, Romania; eugen.tieranu@umfcv.ro; 8 Department of Pulmonology, Faculty of Medicine, Titu Maiorescu University, 031593 Bucharest, Romania; mara.balteanu@prof.utm.ro; 9 Laboratory of Human Genomics, University of Medicine and Pharmacy of Craiova, 200638 Craiova, Romania; anca.costache@umfcv.ro

**Keywords:** PRISm, preserved ratio impaired spirometry, LMICs, low- and middle-income countries, cardiovascular risk

## Abstract

Preserved ratio impaired spirometry (PRISm) is increasingly recognized as a clinically important non-obstructive spirometric phenotype associated with excess all-cause, respiratory, and cardiovascular mortality. PRISm is variably defined across studies and should be distinguished from pre-COPD and restrictive spirometric pattern, particularly in LMIC settings where diagnostic context may differ. Although most evidence has been generated in high-income settings, PRISm may be especially relevant in low- and middle-income countries (LMICs), where the phenotype appears to arise within a markedly different exposure environment. Rather than reflecting predominantly the smoking–obesity–metabolic profile commonly described in wealthier populations, PRISm in LMICs may more often emerge from the cumulative effects of tuberculosis, household biomass smoke, ambient particulate air pollution, poverty-related undernutrition, impaired lung growth, and other adverse life-course exposures. These factors may contribute both to low-volume lung-function impairment and to increased cardiovascular risk through shared pathways of chronic low-grade inflammation, immune activation, oxidative stress, endothelial dysfunction, and metabolic dysregulation. In this context, PRISm may represent a measurable interface between environmental and infectious lung injury, social disadvantage, and systemic vascular vulnerability. The emerging literature further suggests that PRISm in LMICs may include distinct leaner, poverty-related, and infection-linked phenotypes that differ from the obesity-associated patterns more often described in high-income cohorts. This perspective has important clinical implications, as PRISm may identify individuals at elevated risk of cardiometabolic comorbidity, heart failure, stroke, and cardiovascular death who may otherwise remain unrecognized within current respiratory care pathways. Although direct causal evidence remains limited, the convergence of epidemiological, mechanistic, and clinical data supports the view that PRISm in LMICs should be considered a meaningful cardiopulmonary risk state rather than a benign spirometric abnormality. Further LMIC-focused longitudinal, mechanistic, and implementation research is needed to refine phenotyping, clarify causal pathways, and inform integrated prevention strategies.

## 1. Introduction

In low- and middle-income countries (LMICs), preserved ratio impaired spirometry (PRISm) may represent a distinct early lung-health phenotype shaped by tuberculosis, particulate exposure, and life-course poverty, with systemic inflammation and cardiometabolic clustering acting as the bridge to cardiovascular disease.

PRISm, pre-COPD (pre-chronic obstructive pulmonary disease), and restrictive spirometric pattern are related but not interchangeable conditions within the spectrum of non-obstructive lung-function impairment. For clarity, their formal definitions are summarized in Table 1. In practice, PRISm is not defined identically across studies, with variability in the use of fixed-ratio versus lower-limit-of-normal criteria, FEV_1_ thresholds, and pre- versus post-bronchodilator spirometry [1]. These differences influence prevalence estimates, phenotype composition, and comparability across cohorts, and are particularly relevant in LMIC settings, where younger populations, variable reference equations, incomplete post-bronchodilator testing, and overlap with low-FVC phenotypes may alter classification. Current GOLD guidance also distinguishes PRISm from pre-COPD, emphasizing that PRISm should be recognized as a separate spirometric phenotype that may overlap with, but is not subsumed under, pre-COPD. Both constructs may identify individuals at increased risk of later airflow obstruction and therefore have implications for early recognition and follow-up [2,3].

PRISm has emerged as an important non-obstructive spirometric phenotype associated with increased all-cause, respiratory, and cardiovascular mortality. In LMICs, this phenotype may be particularly relevant because it appears to reflect a pattern of lung-function impairment shaped less by the conventional smoking–obesity profile described in high-income settings and more by tuberculosis, household and ambient particulate exposure, and poverty-related life-course insults [9]. These exposures may impair lung growth, promote restrictive or mixed patterns of lung injury, and contribute to PRISm through recurrent infection, chronic inhalational injury, undernutrition, and structural lung damage. Importantly, the same exposures are also linked to increased cardiovascular risk through overlapping mechanisms, including systemic inflammation, oxidative stress, endothelial dysfunction, immune activation, and metabolic dysregulation [6]. Viewed in this way, PRISm may represent a clinically relevant LMIC-specific phenotype at the intersection of environmental and infectious lung injury, social disadvantage, and systemic vascular risk. This review focuses on PRISm because it is a reproducible spirometric phenotype, appears to be common in LMICs, and has been consistently associated with increased cardiovascular morbidity and mortality, including major adverse cardiovascular events, heart failure, stroke, and cardiovascular death [10].

This review aims to synthesize current evidence on the contribution of tuberculosis, particulate exposure, and poverty-related life-course disadvantage to PRISm, and to explore the mechanisms through which these factors may converge to increase cardiovascular risk in LMICs.

## 2. Search Strategy

To enhance transparency and reproducibility, we conducted a targeted search of PubMed/MEDLINE, Embase, Scopus, and Web of Science for studies published from 2005 through 2025. This narrative review was not prospectively registered in PROSPERO or another review registry. The search was performed in the “Topic” field, encompassing title, abstract, and keywords, and combined controlled vocabulary with free-text terms related to: “PRISm” “preserved ratio impaired spirometry”, “non-obstructive spirometric impairment”, “LMICs”, “TB history”, “post-tuberculosis lung disease”, “latent tuberculosis infection” “malnutrition”, “biomass fuels”, “cardiovascular events”, “cardiovascular risk”.

The literature search was conducted in two bibliographic databases, PubMed/MEDLINE and Web of Science Core Collection, in accordance with PRISMA-ScR recommendations for scoping reviews. The PubMed search was performed on 14 March 2026 and covered the period from 1 January 2000 to 14 March 2026. The Web of Science Core Collection search was performed on 15 March 2026 and covered the period from 1 January 2000 to 31 December 2025, in line with the date-filtering options available on that platform.

**Figure 1:**
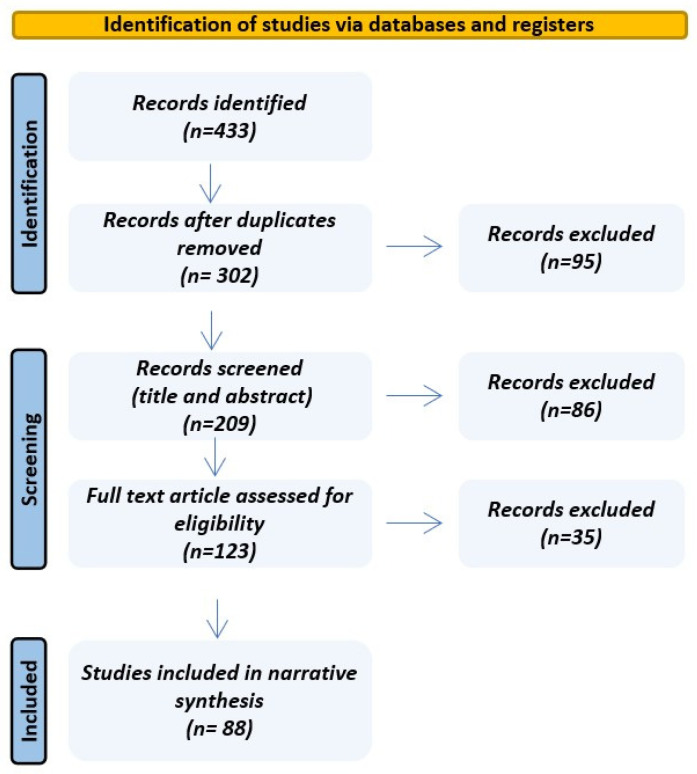
Descriptive flow diagram for study selection.

Only English-language original studies, systematic reviews, meta-analyses, and narrative reviews were considered eligible. Studies were excluded if they lacked sufficient methodological or outcome detail or fell outside the predefined date range. After duplicate removal, titles and abstracts were screened, followed by full-text review to determine final inclusion. A descriptive flow diagram summarizes study selection (Figure 1). Given our narrative aim, evidence was synthesized based on relevance and clinical applicability rather than systematic data extraction; accordingly, no formal risk-of-bias appraisal or quantitative meta-analysis was undertaken.

## 3. PRISm and COPD: Overlap, Divergence, and Transition

PRISm and COPD share several important risk factors and biological pathways, supporting the view that PRISm lies within the broader COPD disease continuum, although it is not equivalent to established COPD. Tobacco smoking remains a major driver of both phenotypes through small-airway injury, chronic inflammation, oxidative stress, and protease–antiprotease imbalance. Similar overlap is seen with ambient particulate pollution and biomass smoke exposure, both of which are associated with impaired lung function, chronic respiratory symptoms, and progression toward obstructive lung disease [11,12,13,14]. Asthma, recurrent respiratory infections, and impaired lung growth further contribute to trajectories that may lead to either PRISm or COPD, highlighting the importance of early-life determinants. In addition, small-airway dysfunction appears to be a shared pathophysiological feature across PRISm, pre-COPD, and early COPD. In LMIC settings, this overlap is likely to be even more complex, as tuberculosis, recurrent infections, poverty-related undernutrition, occupational dusts, and household air pollution add further exposure burdens that may shape both PRISm and COPD. Taken together, these findings support a substantial biological and epidemiological overlap between PRISm and COPD, while still recognizing that PRISm represents a heterogeneous at-risk state rather than established airflow obstruction [15].

COPD and PRISm are defined by different spirometric patterns and should not be used interchangeably. COPD requires persistent post-bronchodilator airflow obstruction, whereas PRISm is characterized by a preserved FEV_1_/FVC ratio with reduced FEV_1_ [1]. Unlike COPD, PRISm is a heterogeneous and unstable phenotype that may reflect low lung volume, small-airway dysfunction, gas trapping, or early airway-predominant disease, and may transition over time to normal spirometry or overt obstruction. This distinction is particularly important in LMICs, where low body mass index (BMI), impaired lung growth, biomass exposure, recurrent infection, and post-tuberculosis lung damage may produce PRISm-like patterns without meeting criteria for classic COPD [8].

Longitudinal evidence indicates that PRISm is an unstable phenotype with multiple possible trajectories, including reversion to normal spirometry, persistence, and progression to airflow obstruction consistent with COPD. Multiple cohorts report that baseline PRISm confers a 2–4-fold increased odds of later airflow obstruction or COPD compared with normal spirometry, including in never-smokers [16,17,18]. In the OLIN study, PRISm at first examination was associated with subsequent obstruction over about a decade (adjusted OR 3.5), with similar effect sizes in current, former, and never-smokers [19]. Parallel analyses in general-population and smoker cohorts show that both PRISm and other pre-COPD indicators (e.g., symptoms, imaging or small-airway abnormalities) follow different lung-function trajectories but each increases future COPD risk. These findings support the view that PRISm is best regarded as a heterogeneous at-risk state rather than a fixed pre-obstructive stage on the COPD continuum [11].

In LMICs, a practical distinction between PRISm and COPD is essential because many symptomatic patients have non-obstructive, low-volume, or post-infectious patterns that do not represent classic COPD. Post-bronchodilator spirometry should be used whenever feasible, as pre- and post-bronchodilator classifications often disagree and pre-bronchodilator PRISm can conceal early obstructive or reversible disease [13]. In addition to confirming persistent airflow obstruction (FEV_1_/FVC < 0.70) for COPD, clinicians should systematically evaluate FVC (and, where available, total lung capacity), symptom burden, and detailed exposure history (tobacco, biomass, ambient pollution) [15]. BMI and nutritional status, prior tuberculosis or HIV, and radiologic or physiological evidence of small-airway dysfunction, gas trapping, or reduced lung volumes can help identify PRISm subtypes such as low-volume or post-infectious phenotypes that are at risk but distinct from overt obstruction [20]. This more nuanced approach is particularly important in LMICs, where post-TB damage, impaired peak lung growth, and poverty-related undernutrition can generate PRISm-like patterns that might otherwise be misclassified as early COPD or generic “restrictive” disease.

## 4. PRISm as a Cardiovascular Risk Phenotype: The Short Recap

PRISm is now firmly established as an adverse prognostic phenotype rather than a benign spirometric oddity. Multiple large cohorts and recent meta-analyses show that, compared with normal spirometry, PRISm carries consistently higher risks of all-cause mortality (pooled HR ≈ 1.6–1.7) [6], cardiovascular mortality (HR ≈ 1.6–1.9), and respiratory mortality (HR ≈ 2–3, reaching >5 in some analyses) [6,7,8,10]. Population studies from the UK Biobank, NHLBI Pooled Cohorts, and other longitudinal cohorts confirm excess coronary heart disease events, stroke, heart failure, and respiratory hospitalizations in PRISm even after adjustment for smoking, obesity, and comorbidities [21,22]. Recent synthesis work extends this picture: systematic reviews and meta-analyses demonstrate strong associations of PRISm with diabetes, hypertension, ischemic heart disease, heart failure, and hypercholesterolemia, positioning PRISm squarely within cardiometabolic multimorbidity rather than as an isolated lung function variant. Genetic correlation analyses further link PRISm loci with type 2 diabetes, blood pressure traits, and cardiovascular phenotypes, reinforcing its interpretation as a systemic, vascular-risk signal [23,24].

PRISm should be viewed as a measurable cardiovascular risk phenotype: a spirometric marker that flags heightened vulnerability to all-cause, cardiovascular, and respiratory death, embedded in a broader cardiometabolic disease cluster rather than confined to the respiratory domain. In LMIC settings, where low FEV_1_, low FVC, post-infectious damage, and impaired lung growth may overlap, the broader umbrella term ‘non-obstructive low-volume spirometric phenotypes’ may sometimes be useful to describe these patterns without implying a single mechanism; however, PRISm remains the primary phenotype of focus in this review because it is reproducible and prognostically relevant [25] (Table 2).

## 5. Why the LMIC Context Changes the PRISm Story

Most current concepts of PRISm have been derived from high-income cohorts, where the phenotype is commonly described in relation to smoking, obesity, and cardiometabolic multimorbidity. In UK Biobank, PRISm was strongly associated with obesity, current smoking, asthma, female sex, cardiovascular comorbidity, and excess mortality [27,28]. A 2024 meta-analysis likewise found higher cardiovascular mortality in PRISm than in normal spirometry, but also emphasized that the available outcome literature is drawn predominantly from developed countries, limiting direct extrapolation to LMICs [25].

PRISm should be interpreted as a heterogeneous spirometric state whose clinical significance is shaped by the exposure environment in which it develops, rather than as a single, uniformly expressed pre-obstructive phenotype. Emerging data from LMICs support this view. In the largest LMIC analysis to date, based on population samples from Peru, Nepal, and Uganda, PRISm prevalence varied markedly across sites, ranging from 2.5% in Peru to 9.1% in Nepal and 16.0% in Uganda [9]. Younger age and female sex were associated with higher odds of PRISm, whereas the association with biomass exposure was not consistent across all sites, underscoring substantial context-specific heterogeneity. Sex differences in PRISm risk in LMICs likely reflect both biological and exposure-related factors. Studies from Uganda and Nigeria suggest that higher PRISm risk among women may partly be explained by sex-specific exposure environments, particularly household air pollution, indoor cooking, and poor indoor air quality, which disproportionately affect women in many LMIC settings. Consistent with this interpretation, female sex was again associated with higher odds of PRISm among Ugandans evaluated after pneumonia, raising the possibility that sex may act, at least in part, as a proxy for unmeasured particulate and household exposure burdens.

These findings suggest that the determinants of PRISm in LMICs are shaped by local exposure patterns rather than by a fixed smoking–obesity profile [29]. In the Peru, Nepal, and Uganda analysis, female sex independently increased the odds of PRISm, whereas the association with biomass exposure varied across sites, underscoring substantial context-specific heterogeneity [9]. Complementary studies from Uganda and Nigeria further suggest that higher PRISm risk among women may partly reflect greater exposure to household air pollution, indoor cooking, and poor indoor air quality, with female sex potentially acting as a proxy for unmeasured particulate exposure in some LMIC settings [30,31]. Together, these data point toward a leaner, lower-volume, and more infection-linked phenotype than the classic smoking–obesity model.

Emerging evidence from sub-Saharan Africa suggests that non-obstructive spirometric impairment in LMICs may reflect a distinct pathway from that typically described in high-income settings [32]. In urban Malawi, more than 40% of adults had abnormal lung function, predominantly spirometric restriction, in a context of extensive biomass fuel use and high HIV prevalence, with low BMI, rather than obesity, strongly associated with restrictive impairment. Follow-up work in rural Malawi similarly demonstrated markedly reduced FVC in approximately one-third of adults, with deficits already apparent in mid-life and no evidence of accelerated decline compared with healthy high-income cohorts, suggesting that reduced peak lung growth may be more important than accelerated adult loss in shaping these patterns [33].

Among people living with HIV in Kenya, low BMI and previous pulmonary tuberculosis were likewise associated with FVC below the lower limit of normal, reinforcing the contribution of malnutrition and recurrent infectious lung injury to non-obstructive lung-function deficits [34]. These findings differ from the phenotype more often reported in high-income settings, where PRISm and restrictive spirometric pattern are commonly associated with obesity, smoking, asthma, and diabetes [35,36]. In contrast, LMIC cohorts and commentaries increasingly link PRISm and low-FVC phenotypes to lower BMI, biomass exposure, and poverty. This interpretation is consistent with multinational BOLD analyses showing that poverty is associated with lower FEV_1_/FVC and higher odds of chronic airflow obstruction, while greater wealth is associated with better lung function.

Latent tuberculosis infection (LTBI) and prior active tuberculosis are associated with persistent low-grade inflammation and immune activation through pathways that overlap with atherogenesis, including tumor necrosis factor-α, interferons, interleukins, and heat shock protein 65-related autoimmunity [37,38]. LTBI has been linked to higher risks of coronary artery disease and acute myocardial infarction, and has also been associated with subclinical obstructive coronary atherosclerosis in LMIC cohorts [39]. Population studies suggest a modest increase in incident cardiovascular disease and hypertension among individuals with LTBI, particularly when treatment is incomplete. Similarly, meta-analytic data indicate that active or prior tuberculosis is associated with an approximately 1.5-fold higher risk of major adverse cardiovascular events and cardiovascular mortality compared with non-TB populations [40,41]. These findings suggest that tuberculosis may amplify cardiovascular risk through the combined effects of persistent immune activation and residual lung injury, making it a plausible contributor to PRISm-related cardiovascular vulnerability.

Evidence from reviews and cohort studies indicates that tuberculosis survivors remain at increased cardiovascular risk for years after microbiological cure, particularly for atherosclerotic events such as myocardial infarction, stroke, and peripheral arterial disease [42]. Recent data further suggest that latent and active tuberculosis may act as non-traditional ASCVD risk factors, interacting with diabetes and other cardiometabolic conditions to amplify vascular risk [43]. Post-tuberculosis lung impairment is common, with systematic reviews showing abnormal spirometry in approximately 46–60% of survivors in LMICs, including restrictive and mixed ventilatory defects [44,45]. Persistent reductions in FVC and FEV_1_, together with fibrosis, bronchiectasis, and cavitary damage, indicate a combined structural and functional burden [46].

**Figure 2:**
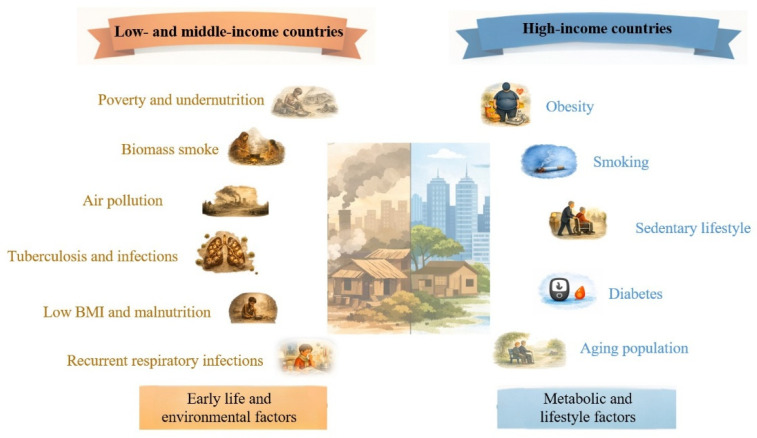
PRISm risk factors: low- and middle-income countries versus high income countries. BMI: body mass index.

These data support the view that PRISm and related non-obstructive phenotypes in LMICs may often arise from adverse life-course conditions, impaired lung development, and cumulative infectious and environmental exposures rather than from the conventional smoking–obesity profile seen in wealthier settings [47,48] (Figure 2).

### 5.1. Tuberculosis and Post-Tuberculosis Lung Disease as Plausible Contributors to PRISm-Related Cardiovascular Risk

Direct evidence that tuberculosis or post-tuberculosis lung disease causes PRISm remains limited. However, tuberculosis and post-tuberculosis lung disease are established markers of elevated cardiovascular risk and persistent structural lung impairment, creating a biologically plausible framework in which they may contribute to PRISm-related cardiovascular vulnerability.

Multiple epidemiologic datasets show that people with prior or current TB have substantially higher 10-year ASCVD risk, incident CVD, MI and stroke than non-TB controls, even after adjustment for traditional risk factors [49,50,51]. In Korea, post-TB survivors had markedly higher odds of being in the high ASCVD risk category (OR 1.69, 95% CI 1.59–1.78) than those without TB [49]. A global meta-analysis estimated a pooled 1.5-fold increase in major adverse cardiac events among people diagnosed with TB [50]. Large US/UK cohort analyses similarly demonstrate 1.6–3.2-fold higher CVD incidence around the time of TB diagnosis, persisting after adjustment for baseline CVD risk [51]. Chronic immune activation may promote atherogenesis through heat shock protein–mediated vascular injury. Under inflammatory or infectious stress, HSP60/65 is upregulated on endothelial cells, macrophages, and smooth muscle cells, where it acts as a danger signal and sustains low-grade vascular inflammation. Anti-HSP65/60 antibodies may then bind stressed endothelial cells and induce complement-dependent or antibody-dependent cellular cytotoxicity, thereby promoting endothelial injury, intimal infiltration, and plaque formation. In addition, antibodies against mycobacterial HSP65 can cross-react with human HSP60 epitopes in early arterial lesions, supporting a mechanism of molecular mimicry. Elevated anti-mHSP65 titers have also been associated with carotid atherosclerosis and coronary calcification, consistent with pathogen-triggered autoimmunity as a contributor to vascular disease [52,53].

TB frequently leaves residual structural lung damage such as fibrosis, bronchiectasis, volume loss, and post-TB survivors have an excess burden of chronic respiratory impairment and other chronic diseases compared with the general population [49,54]. Conceptually, this pattern of reduced lung volumes and airway–parenchymal distortion overlaps with spirometric phenotypes characterized by low FVC and non-obstructive limitation, which are strongly linked to cardiometabolic multimorbidity and CVD in non-TB populations.

However, direct evidence that TB or post-TB lung disease causes PRISm is sparse. Existing TB-CVD studies do not routinely report detailed spirometry or classify post-TB survivors into PRISm versus other lung function patterns [49,50,51]. Systematic reviews of TB and CVD explicitly note that current data support TB as a marker of higher CVD risk but are insufficient for firm causal inferences or for disentangling lung-function–mediated pathways.

The hypothesis that TB and post-tuberculosis lung disease contribute to PRISm-related cardiovascular risk is currently supported by triangulated evidence. TB induces chronic systemic inflammation and metabolic perturbations that may promote atherosclerosis and cardiometabolic risk [55,56]. It also frequently results in persistent lung impairment, including low-volume and mixed ventilatory defects that may overlap with PRISm. In parallel, PRISm has been independently associated with cardiometabolic clustering and excess cardiovascular events in population-based studies. Together, these findings provide a plausible mechanistic basis for viewing TB and post-TB lung disease as contributors to PRISm-associated cardiovascular vulnerability [57].

Given the overlap between post-TB low-volume spirometric patterns and the PRISm phenotype, it is plausible that a subset of TB survivors falls into a PRISm trajectory, amplifying cardiometabolic and vascular risk. However, TB-to-PRISm pathways remain largely inferential, as most TB–CVD cohorts lack detailed spirometric phenotyping. Prospective studies with standardized lung function, imaging, and cardiovascular outcomes are needed to confirm whether post-TB PRISm constitutes a distinct high-risk endotype.

### 5.2. Household Air Pollution, Biomass Smoke, and Ambient Particulate Matter

#### 5.2.1. Household Air Pollution and Biomass Smoke

Household air pollution from biomass combustion is pervasive in LMICs and is a plausible upstream contributor to PRISm-related cardiovascular risk, although direct PRISm-specific evidence remains limited. Reliance on solid fuels for cooking and heating exposes billions of people to very high levels of PM_2.5_ and other toxic co-pollutants, contributing to COPD, chronic bronchitis, and other chronic lung conditions. These exposures often produce predominant airway involvement with less emphysema, a pattern compatible with low-volume, non-obstructive spirometric phenotypes such as PRISm [58,59].

#### 5.2.2. Ambient Particulate Pollution

Ambient particulate matter, particularly PM_2.5_ and smaller fractions, is also strongly associated with increased cardiovascular morbidity and mortality in LMICs, including stroke, heart failure, hypertension, and ischemic heart disease. Long-term particulate exposure may confer even greater excess cardiovascular risk in LMIC settings than in many high-income populations, likely reflecting higher exposure levels and background vulnerability [60,61].

#### 5.2.3. Shared Cardiometabolic Mechanisms

Mechanistically, both biomass smoke and ambient particulate pollution promote oxidative stress, pulmonary and systemic inflammation, endothelial dysfunction, autonomic imbalance, metabolic disturbance, atherosclerosis, and thrombosis, thereby contributing to both structural lung injury and cardiometabolic derangements [62,63]. In LMIC populations, where personal PM_2.5_ exposures are often highest and frequently dominated by household biomass sources, the convergence of impaired lung development or chronic lung injury with pollution-induced cardiometabolic injury provides a biologically coherent pathway to a PRISm phenotype clustered with elevated cardiovascular risk, even if PRISm itself has not yet been systematically characterized in these exposure cohorts [58,64].

### 5.3. Poverty, Undernutrition, Impaired Lung Growth, and the Low-BMI PRISm Phenotype

Poverty, undernutrition, and impaired lung growth in LMICs create a distinct low-BMI PRISm phenotype that is tightly intertwined with elevated cardiovascular risk, even if the PRISm label is rarely applied in existing cohorts. In LMIC spirometry surveys, PRISm and spirometric restriction are both highly prevalent and paradoxically associated with lower BMI and poverty, rather than obesity, suggesting a “thin, low-volume” phenotype linked to deprivation [9,10]. Longitudinal birth-cohort data show that maternal nutritional problems, being born small for gestational age, and being underweight in childhood all independently predict adult spirometric restriction (low FVC with preserved ratio), with effects largely mediated by low lean mass [65]. This restrictive pattern is strongly associated with increased all-cause, cardiovascular, and respiratory mortality in adulthood. Mechanistically, chronic undernutrition and stunting slow lung growth and reduce alveolarization, leading to smaller lungs and proportionally reduced FEV_1_ and FVC but preserved FEV_1_/FVC (“lung-sparing growth”) [66]. In LMICs, low lung capacity is increasingly recognized as analogous to stunting, sharing early-life nutritional and inflammatory drivers and acting as a powerful predictor of later cardiovascular mortality. Severe childhood undernutrition or famine exposure also tracks forward to higher rates of adult cardiovascular disease, hypertension, diabetes and metabolic syndrome, indicating that poverty-related nutritional insults program both impaired lung capacity and cardiometabolic vulnerability [67]. Within this context, low-BMI PRISm in LMICs can be framed as a syndemic phenotype in which poverty, early undernutrition, constrained lung growth and reduced lean mass converge to produce a restrictive spirometric pattern that clusters with systemic inflammation and cardiometabolic risk, thereby amplifying PRISm-related cardiovascular risk despite the absence of classical obesity-driven mechanisms [67].

## 6. PRISm as a Cardiovascular Risk Phenotype in LMICs: Beyond the Obesity–Smoking Model

Large UK Biobank and Rotterdam/UK studies show PRISm associated with higher major adverse cardiovascular events, heart failure, stroke, and cardiovascular mortality, even after extensive adjustment [22,24]. A pooled US cohort analysis (NHLBI Pooled Cohorts) found higher CHD (coronary heart disease) mortality and CHD events in PRISm vs normal spirometry [23]. Meta-analytic work shows strong associations between PRISm and diabetes, hypertension, ischemic heart disease, heart failure, and higher BMI, reinforcing a cardiometabolic clustering in many HIC populations [68].

However, the mechanisms underlying PRISm in LMICs are unlikely to be identical. In BOLD and Malawian cohorts, PRISm and related low-volume phenotypes are more often associated with lower BMI and poverty than with obesity [68]. These observations support a dual-pathway model in which the classic metabolic–obesity pathway coexists with a low-volume poverty–injury pathway, particularly relevant in LMICs, where poverty, undernutrition, household biomass exposure, ambient particulate pollution, and recurrent infections may impair lung growth and reduce lung volumes while simultaneously increasing cardiovascular risk through chronic inflammation and social disadvantage [69,70]. This contrast should be interpreted as a difference in dominant patterns rather than a strict dichotomy. PRISm in high-income settings is not confined to obese individuals, and obesity-associated PRISm may also be present in LMICs, particularly in populations undergoing nutritional transition and increasing cardiometabolic burden. The available data therefore support heterogeneity within both settings, with variation in the relative contribution of adiposity, impaired lung growth, infection, and environmental exposure [68].

## 7. Inflammation as the Mechanistic Bridge

**Figure 3:**
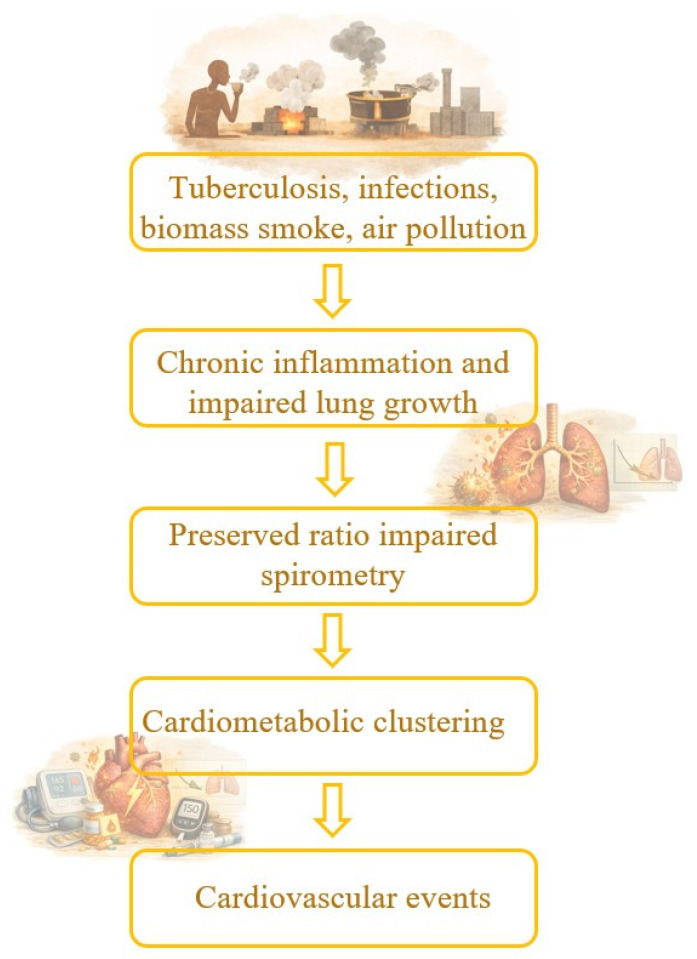
Conceptual pathway linking LMIC-specific exposures, PRISm, and cardiovascular events.

Chronic low-grade inflammation appears to be a central mechanism linking PRISm with cardiovascular risk. Elevated hs-CRP and composite inflammatory scores are associated with higher fasting glucose, blood pressure, dyslipidemia, diabetes, hypertension, and incident cardiovascular events and mortality [71,72] (Figure 3).

In LMIC PRISm cohorts, elevated hs-CRP and greater glucose intolerance have been reported independently of obesity, supporting the concept of a lung–systemic inflammatory phenotype rather than a purely mechanical reduction in lung volume [10]. Similar pathways are relevant to major LMIC exposures. LTBI is characterized by persistent immune activation, including monocyte/macrophage activation, increased pro-inflammatory cytokines, and vascular trafficking of inflammatory cells [73,74], with proposed mechanisms involving sustained inflammatory signaling, autoimmunity to mycobacterial heat shock proteins, and possible pathogen persistence within atherosclerotic plaques [41,75]. Epidemiological evidence supports this link, suggesting that LTBI is associated with roughly doubled odds of coronary artery disease or myocardial infarction, as well as a higher incidence of hypertension and related cardiometabolic risk factors. Long-term exposure to PM_2.5_ [56,57] and ultrafine particulate matter also promotes oxidative stress, endothelial dysfunction, autonomic imbalance, insulin resistance, dyslipidemia, and metabolic syndrome, thereby increasing cardiovascular incidence and mortality, including myocardial infarction and stroke [73,74]. Taken together, PRISm, tuberculosis-related immune activation, and long-term particulate exposure appear to converge on chronic inflammation and cardiometabolic dysregulation, fostering low-volume lung phenotypes, clustering of diabetes and hypertension, and ultimately excess cardiovascular events [55,61] (Table 3).

## 8. Clinical Implications for LMIC Practice

PRISm is common in LMICs and is associated with increased all-cause, cardiovascular, and respiratory mortality; it should therefore not be regarded as a benign or borderline spirometric abnormality [6]. In routine practice, this has important implications for both respiratory and cardiovascular care, particularly in health systems where chronic respiratory disease remains underdiagnosed and cardiovascular risk assessment is inconsistently implemented.

In practice, PRISm is unlikely to be identified through stand-alone screening programs in many LMIC settings, because spirometry is often unavailable in primary care and respiratory services are concentrated in referral centers [6]. A more feasible approach may be opportunistic case-finding embedded within existing care pathways, particularly tuberculosis follow-up clinics, HIV clinics, chronic respiratory disease services, and non-communicable disease clinics managing hypertension or diabetes [41]. In such settings, symptomatic individuals or those with major exposure histories could undergo targeted spirometry where available, while peripheral facilities could use symptom screening, exposure history, pulse oximetry, and referral algorithms linked to district-level spirometry hubs.

At the same time, caution is warranted to avoid over-medicalizing PRISm in resource-limited settings where spirometry quality may be variable and phenotype boundaries remain uncertain [11]. Potential harms include misclassification, unnecessary anxiety, inappropriate inhaler prescribing, and diversion of scarce resources away from higher-priority interventions [23]. Direct cost-effectiveness data for PRISm-specific screening in LMICs are currently lacking; accordingly, integrating PRISm assessment into existing tuberculosis, HIV, and cardiovascular risk pathways is likely to be more pragmatic than broad population-based spirometry screening until stronger implementation evidence becomes available [76].

A first priority is to improve case finding and risk stratification. LMIC health systems should move beyond a narrow focus on overt chronic obstructive pulmonary disease and instead systematically recognize and act on all abnormal spirometric patterns, including PRISm, given its prevalence, symptom burden, and prognostic significance. Individuals with PRISm should be considered a high-risk cardiometabolic group and undergo routine assessment for hypertension, diabetes, dyslipidemia, and heart failure, all of which appear to be more common in this phenotype [6,10]. In settings where access to laboratory testing is limited, simplified non-laboratory cardiovascular risk tools and blood pressure-based treatment algorithms may provide a pragmatic way to integrate PRISm into existing WHO PEN or primary-care cardiovascular risk assessment pathways [77].

Management priorities in resource-constrained settings should focus on low-cost interventions that address shared cardiometabolic risk. Evidence from general PRISm cohorts suggests that better overall cardiovascular health is associated with substantially lower cardiovascular and mortality risk and may also favor transition from PRISm to normal spirometry [78]. This supports prioritizing tobacco cessation, blood pressure control, diabetes management, weight and nutritional optimization, and physical activity using protocolized primary-care packages that are feasible in LMIC settings [60]. Community-based and task-shifted models, including nurse-led or community health worker-led hypertension programs, mobile health support, and group education strategies, may be especially useful for PRISm-flagged patients because such models have already shown benefit in improving blood pressure control and cardiovascular care delivery in low-resource settings [79]. In addition, the persistently low use of statins in LMICs represents an important missed opportunity; PRISm may serve as an additional clinical cue to intensify lipid-lowering and antihypertensive treatment in eligible individuals.

At the health-system level, PRISm should be explicitly incorporated into LMIC respiratory and cardiovascular guidelines through simple spirometry interpretation pathways linked to integrated cardiovascular risk assessment and management packages. At the same time, further LMIC-based cohort studies are needed to distinguish low-BMI and obesity-related PRISm phenotypes, clarify the contribution of environmental and infectious exposures, and identify interventions that are both effective and scalable in different resource settings [77].

## 9. Research and Policy Agenda

Although PRISm appears to be common in LMICs, it remains poorly characterized, as most longitudinal, mechanistic, and interventional evidence still comes from high-income cohorts [10,26]. The next phase of research should move beyond prevalence estimates toward a more precise understanding of exposure-specific pathways, phenotype heterogeneity, cardiovascular consequences, and pragmatic interventions in LMIC settings.

A first priority is the development of LMIC-based longitudinal cohorts with repeated spirometry to define transitions between normal lung function, PRISm subtypes, and airflow obstruction. These studies should explicitly measure LMIC-relevant exposures, including early-life undernutrition, poverty, household biomass use, ambient air pollution, farming and organic dust exposure, and recurrent infections, in order to clarify their contribution to incident PRISm and its trajectories [10,24]. A second priority is improved phenotyping. PRISm should be stratified into clinically meaningful subtypes, including restrictive versus non-restrictive and low-BMI versus obesity-related forms, and linked to inflammatory, metabolic, and imaging profiles to determine whether distinct biological phenotypes exist in LMIC populations [6,80].

A third priority is replication of large-scale cardiovascular outcome analyses in LMICs. Studies should assess whether PRISm predicts heart failure, stroke, major adverse cardiovascular events, and cardiovascular mortality in these settings, and whether low-volume or restrictive phenotypes carry disproportionate risk [1,21]. Integrating biomarkers of systemic inflammation, immune activation, and metabolic dysfunction will be important for testing the hypothesis that PRISm represents an early cardiopulmonary risk state rather than only a spirometric category [6,81]. A fourth priority is intervention and implementation research. PRISm case-finding should be incorporated into existing chronic respiratory and cardiovascular care platforms, with pragmatic evaluation of smoking cessation, blood pressure and lipid control, diabetes care, nutrition, and physical activity interventions, as well as low-cost spirometry deployment and task-sharing approaches in primary care.

PRISm should be explicitly recognized within LMIC respiratory and cardiovascular strategies as a distinct non-obstructive spirometric phenotype with prognostic and management implications [9].

Testing the distinct-phenotype hypothesis will require multicountry longitudinal cohorts with harmonized spirometry, body-composition assessment, imaging, inflammatory phenotyping, and detailed exposure quantification. Approaches such as latent class analysis, trajectory modeling, and stratification by nutritional transition may help determine whether low-BMI and obesity-associated PRISm represent distinct biological endotypes or overlapping variants along a shared continuum.

Greater investment in LMIC-led longitudinal cohorts, surveillance systems, and integrated evidence-to-policy platforms will be essential to generate context-specific data and translate it into respiratory and cardiovascular prevention strategies.

## 10. Limitations

The available literature should be interpreted within the context of several methodological considerations. Most data linking PRISm to cardiovascular outcomes originate from high-income cohorts, while comparable evidence from LMICs is still emerging. Moreover, relatively few studies have integrated spirometry, infectious and environmental exposures, inflammatory profiling, and adjudicated cardiovascular endpoints in the same population over time. The proposed contribution of tuberculosis, particulate exposure, and poverty-related life-course disadvantage to PRISm-related cardiovascular risk therefore rests largely on converging lines of evidence rather than direct causal demonstration. In addition, differences in PRISm definitions across studies, including the use of fixed-ratio versus lower-limit-of-normal criteria, variation in FEV_1_ thresholds and bronchodilator protocols, as well as overlap with restrictive spirometric phenotypes and variable spirometry quality, may partly account for heterogeneity across studies and should be considered when interpreting the literature.

## 11. Conclusions

PRISm is increasingly recognized as a clinically important non-obstructive spirometric phenotype associated with cardiovascular, respiratory, and all-cause mortality. In LMICs, it may represent a distinct phenotype shaped less by the conventional smoking–obesity profile described in high-income settings and more by tuberculosis, particulate exposure, poverty-related undernutrition, impaired lung growth, and other adverse life-course insults. These exposures likely converge through chronic inflammation, immune activation, oxidative stress, and cardiometabolic dysregulation, linking low-volume lung-function impairment to increased cardiovascular risk. Although direct causal evidence remains limited, the available literature supports the view that PRISm should be regarded as a meaningful cardiopulmonary risk state rather than a benign spirometric abnormality. Greater recognition of PRISm in LMIC research, clinical care, and policy may improve risk stratification and help develop more integrated strategies for the prevention of both respiratory and cardiovascular disease.

## Figures and Tables

**Table 1:** Diagnostic boundaries of related non-obstructive spirometric constructs.

Construct	Short Definition	Spirometric Basis	Key Distinction
Pre-COPD	Respiratory symptoms, structural abnormalities, or physiological abnormalities suggestive of early COPD, but without persistent airflow obstruction	FEV_1_/FVC not obstructed; abnormalities may include symptoms, imaging changes, reduced diffusion, small-airway dysfunction, or accelerated decline [4]	A broader clinical construct; not defined by one spirometric pattern alone
PRISm	Preserved FEV_1_/FVC ratio with reduced FEV_1_	FEV_1_/FVC ≥ 0.70 or ≥LLN, with FEV_1_ < 80% predicted or <LLN [5]	A distinct spirometric phenotype associated with heterogeneous mechanisms and outcomes
Restrictive spirometric pattern	Preserved FEV_1_/FVC ratio with reduced FVC	FEV_1_/FVC preserved, with FVC < 80% predicted or <LLN [6]	Overlaps partly with PRISm but is centered on low FVC rather than low FEV_1_
COPD	Persistent airflow limitation, usually associated with chronic respiratory symptoms and exposure history	Post-bronchodilator FEV_1_/FVC < 0.70 or below LLN, depending on framework used [7,8]	Defined by obstruction, unlike PRISm and restrictive spirometric pattern

PRISm, preserved ratio impaired spirometry; LLN, lower limit of normal; FEV_1_, forced expiratory volume in 1 s; FVC, forced vital capacity; COPD, chronic obstructive pulmonary disease.

**Table 2:** Spirometric phenotypes, overlap, and cardiovascular relevance.

Phenotype	Core Spirometric Pattern	Relation to PRISm	Relation to COPD	Cardiovascular Relevance	LMIC Relevance
Pre-COPD	No persistent airflow obstruction; may include symptoms, imaging abnormalities, reduced diffusion, or small-airway dysfunction [5]	May overlap with PRISm, but is broader and not defined by a single spirometric pattern	Represents an at-risk state for later airflow obstruction	Cardiovascular risk is not defined by spirometry alone, but may coexist through shared inflammatory and exposure-related pathways	Particularly relevant where symptoms, prior infection, and exposure burden are high but obstruction is absent
PRISm	Preserved FEV_1_/FVC with reduced FEV_1_	Primary phenotype of interest	Distinct from COPD, but may transition to COPD, persist, or revert to normal spirometry [4]	Strongly associated with increased all-cause, respiratory, and cardiovascular mortality, as well as cardiometabolic multimorbidity	Common in LMICs; may reflect tuberculosis, particulate exposure, poverty-related undernutrition, impaired lung growth, and other life-course insults
Restrictive spirometric pattern	Preserved FEV_1_/FVC with reduced FVC	Partly overlaps with PRISm, especially in low-volume phenotypes [1,11]	Not COPD because obstruction is absent	Associated with adverse outcomes, including increased mortality, but cardiovascular relevance may differ from PRISm	Important in LMICs because low BMI, malnutrition, post-infectious lung damage, and impaired peak lung growth may produce low-FVC patterns [21,22]
COPD	Persistent post-bronchodilator airflow obstruction	May arise from a subset of PRISm trajectories, but is not interchangeable with PRISm [26]	Established obstructive lung disease	Associated with substantial cardiovascular comorbidity and mortality	In LMICs, COPD is shaped not only by smoking but also by biomass smoke, household air pollution, occupational dusts, and prior tuberculosis
Non-obstructive low-volume spirometric phenotypes	Preserved ratio with reduced FEV_1_ and/or FVC, often without classic obstruction	Umbrella category that may include PRISm-like and restrictive-like patterns	Distinct from COPD, although some individuals may later progress to obstruction	Likely relevant to cardiovascular risk through shared inflammatory, developmental, and exposure-related mechanisms [25]	Particularly useful as a descriptive umbrella term in LMICs, where post-TB damage, undernutrition, impaired lung growth, and mixed low-volume patterns may overlap

PRISm, preserved ratio impaired spirometry; COPD, chronic obstructive pulmonary disease; FEV_1_, forced expiratory volume in 1 s; FVC, forced vital capacity.

**Table 3:** Evidence map for proposed PRISm-related cardiovascular pathways in LMICs.

Pathway/Exposure	Established Association	Plausible Mechanism	Direct PRISm-Specific Evidence	Key Evidence Gap	Overall Strength of Evidence
Tuberculosis/post-TB lung disease	Prior or active TB is associated with increased ASCVD risk, incident cardiovascular disease, myocardial infarction, stroke, and cardiovascular mortality; post-TB lung impairment is common and often includes restrictive or mixed ventilatory defects [55,61]	Chronic immune activation, endothelial injury, metabolic perturbation, residual fibrosis/bronchiectasis/volume loss, and overlap with low-volume non-obstructive spirometric patterns	Low	Few studies classify post-TB survivors specifically as PRISm versus other non-obstructive phenotypes; longitudinal TB–PRISm–CVD pathways remain largely inferential	Moderate
LTBI and immune activation	LTBI has been associated with coronary artery disease, myocardial infarction, subclinical coronary atherosclerosis, hypertension, and cardiometabolic risk [73,74]	Persistent low-grade inflammation, monocyte/macrophage activation, cytokine signaling, endothelial dysfunction, and possible molecular mimicry involving mycobacterial heat shock proteins	Low	Direct evidence linking LTBI to PRISm is sparse; most data relate LTBI to cardiovascular outcomes rather than spirometric phenotypes	Moderate
Household biomass exposure	Biomass smoke is associated with chronic respiratory symptoms, COPD, chronic bronchitis, and abnormal lung-function patterns in LMICs; household air pollution is also linked to cardiovascular morbidity and mortality [58,59]	Chronic inhalational injury, oxidative stress, pulmonary and systemic inflammation, endothelial dysfunction, autonomic imbalance, and promotion of low-volume airway-predominant lung injury	Low to moderate	PRISm has not been systematically characterized in most biomass-exposed cohorts; uncertainty remains regarding phenotype specificity	Moderate
Ambient particulate pollution	Long-term PM exposure is strongly associated with cardiovascular morbidity and mortality in LMICs, including stroke, heart failure, hypertension, and ischemic heart disease [60,61]	Oxidative stress, systemic inflammation, endothelial dysfunction, autonomic dysregulation, insulin resistance, dyslipidemia, atherosclerosis, and thrombosis	Low	Direct LMIC studies linking ambient PM specifically to PRISm are limited; most evidence supports CVD risk more strongly than PRISm classification	Moderate to high
Poverty/undernutrition/impaired lung growth	Low BMI, poverty, and low-FVC or restrictive-like phenotypes are common in LMIC cohorts; early-life undernutrition is linked to smaller adult lung volumes and later cardiometabolic disease [9,10]	Impaired peak lung growth, reduced alveolarization, reduced lean mass, chronic low-grade inflammation, developmental programming of later cardiometabolic vulnerability	Moderate	Need clearer distinction between low-BMI PRISm, restrictive spirometric pattern, and other non-obstructive low-volume phenotypes in LMIC cohorts	Moderate
PRISm and cardiovascular outcomes	PRISm is consistently associated with higher all-cause, respiratory, and cardiovascular mortality, as well as diabetes, hypertension, ischemic heart disease, and heart failure [22,24]	Shared inflammatory and cardiometabolic pathways, small-airway dysfunction, low-volume phenotypes, and systemic vascular vulnerability	High for general populations; low to moderate for LMIC-specific cohorts	Most outcome data come from high-income populations; LMIC-specific longitudinal CVD analyses remain limited	High overall; Moderate in LMICs

## Data Availability

The original contributions presented in this study are included in the article. Further inquiries can be directed to the corresponding authors.
